# Cancer stem cells and their niche in cancer progression and therapy

**DOI:** 10.1186/s12935-023-03130-2

**Published:** 2023-12-01

**Authors:** Qiuping Liu, Zongliang Guo, Guoyin Li, Yunxia Zhang, Xiaomeng Liu, Bing Li, Jinping Wang, Xiaoyan Li

**Affiliations:** 1 Institute of Translational Medicine, College of Life Science and Agronomy, Zhoukou Normal University, Zhoukou, 466001 Henan China; 2 Department of General Surgery, Shanxi Province Cancer Hospital, Affiliated of Shanxi Medical University, Taiyuan, 030013 Shanxi China; 3 Department of Ultrasound, Shanxi Province People’s Hospital, Taiyuan, 030012 Shanxi China; 4 Department of blood transfusion, Shanxi Provincial People’s Hospital, Taiyuan, 030032 Shanxi China; 5 Department of central laboratory, Shanxi Provincial People’s Hospital, Taiyuan, 030032 Shanxi China

**Keywords:** cancer stem cells, CSC niche, Cellular components, Extracellular matrix, Therapeutic strategies

## Abstract

High recurrence and metastasis rates and poor prognoses are the major challenges of current cancer therapy. Mounting evidence suggests that cancer stem cells (CSCs) play an important role in cancer development, chemoradiotherapy resistance, recurrence, and metastasis. Therefore, targeted CSC therapy has become a new strategy for solving the problems of cancer metastasis and recurrence. Since the properties of CSCs are regulated by the specific tumour microenvironment, the so-called CSC niche, which targets crosstalk between CSCs and their niches, is vital in our pursuit of new therapeutic opportunities to prevent cancer from recurring. In this review, we aim to highlight the factors within the CSC niche that have important roles in regulating CSC properties, including the extracellular matrix (ECM), stromal cells (e.g., associated macrophages (TAMs), cancer-associated fibroblasts (CAFs), and mesenchymal stem cells (MSCs)), and physiological changes (e.g., inflammation, hypoxia, and angiogenesis). We also discuss recent progress regarding therapies targeting CSCs and their niche to elucidate developments of more effective therapeutic strategies to eliminate cancer.

## Introduction

Malignant tumours are diseases that seriously threaten human life. For most patients with malignant tumours, chemotherapy, radiation therapy and biological immunotherapy can be used to kill most of the tumour cells, but they cannot fundamentally cure the tumour. The development of the cancer stem cell (CSC) theory enables life scientists to think about cancer in a new way, helps them to uncover the nature of cancer, and makes a cure for cancer possible [1, 2]. In recent years, the CSC theory has attracted increasing attention, and CSCs have been successfully isolated from various malignant tumours, such as breast cancer, brain tumours, prostate cancer, lung cancer, liver cancer, colorectal cancer, and skin cancer [3, 4]. CSCs are subsets of cells with strong proliferative capacity and high self-renewal and differentiation potential in malignant tumour tissues and are also the root of cancer recurrence and metastasis [5]. In addition, studies have confirmed that CSCs play a decisive role in cancer development, chemoradiotherapy resistance, recurrence and metastasis [6, 7]. Therefore, CSCs are a pivotal target for the eradication of cancers.

Just as cancer cells are regulated by their specific microenvironments, CSCs are also understood to exist in a specific microenvironment, namely, the “CSC niche” or “CSC microenvironment” [8]. The status of CSCs in the primary tumour and the malignant phenotype of their progeny are controlled by various factors generated by the associated CSC niche during tumour progression to a malignant state [9, 10]. The expression of stem cell markers in cancer (stem) cells and their tolerance to anticancer drugs are determined by specific combinations of microenvironmental components [11, 12]. Multiple studies have supported the idea that the reciprocal interaction between CSCs and their putative niches is a crucial component of tumour growth and progression [13–15]. Understanding the mechanism of interaction between CSCs and the CSC niche will likely facilitate the development of effective cancer treatments.

**Fig. 1:**
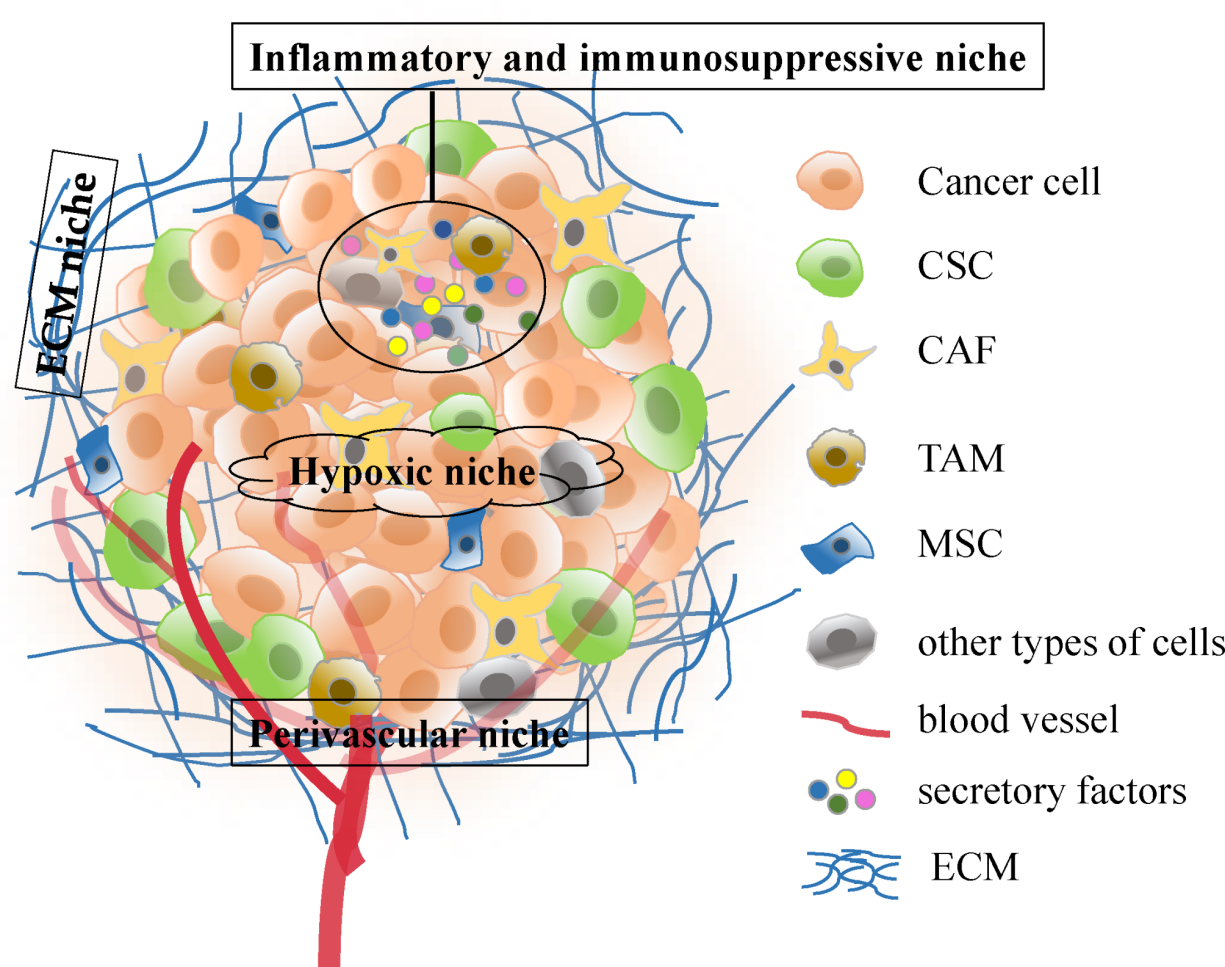
General overview of the components of the CSC niche. Cellular components such as CAFs, TAMs and MSCs and reactions such as inflammation, hypoxia, angiogenesis, and the ECM of the CSC niche promote and support the properties of CSCs. CSC: cancer stem cell, CAF: cancer-associated fibroblast, ECM: extracellular matrix, MSC: mesenchymal stem cells, TAM: tumour-associated macrophages

The CSC niche is a specific tumour microenvironment that supports CSC self-renewal, proliferation, and function. It consists mainly of stromal cells, extracellular matrix (ECM), a variety of cytokines and growth factors [8, 16]. In the CSC niche, reactions such as inflammation, epithelial-mesenchymal transformation (EMT), hypoxia, acidic pH, and angiogenesis constantly occur to keep the internal environment stable. Studies have shown that establishing CSC niches in distant locations is critical for CSC survival and self-renewal [17]. In addition, interactions with adjacent cells in the CSC niche as well as the stroma have been shown to be important for the survival and maintenance of CSCs [18]. The components of the CSC niche and biological processes within it determine the fate of CSCs (Fig. 1). Evidence suggests that the CSC microenvironment plays a crucial role in regulating the properties of CSC, thereby promoting tumorigenesis, progression, treatment resistance, and metastasis [19]. In fact, CSCs also regulate their microenvironment to maintain their properties [20, 21]. The crosstalk between CSC and their microenvironment plays a key role in tumour progression. In this review, we focus on some key factors in CSC niche that play important roles in regulating CSC properties and tumour progression.

## Properties of cancer stem cells

CSCs, also known as tumour-initiating cells (TICs), are elucidated as a distinct population that persist within tumours. These cells are responsible for cancer recurrence, metastasis, and resistance to current therapies, [22–24] based on several of their properties. Firstly, CSCs have the ability to self-renew. In the self-renewal process, CSCs may undergo symmetric or asymmetric division to both maintain a defined CSC population and expand the bulk of tumour [23, 25, 26]. Previous literature has shown that csc self-renew depends on the activation of specific stem cell pathways and inactivation of pathways that inhibit stem cell self-renewal [24, 27]. Secondly, CSCs have differentiation ability. CSCs exhibit multi-differentiation potential to differentiate into cancer cells and a variety of stromal cells, maintaining the CSC microenvironment to promote CSC properties and tumour development. CSCs can differentiate into cancer cells, which has been validated in other types of cancers, including pancreatic, prostate, lung and liver cancer [28]. Tang and colleagues showed that ovarian CSCs differentiate into endothelial cells (ECs) and promote tumour angiogenesis through autocrine C-C Motif Chemokine Ligand 5 (CCL5) signalling [29]. Previous studies have demonstrated that CSCs can differentiate into pericytes via regulating by cell-intrinsic or microenvironmental cues [30, 31]. In addition, it has also been demonstrated that GSCs can differentiate into tumour-associated macrophages (TAMs), cancer-associated fibroblasts (CAFs) or myeloid-derived suppressor cells (MDSCs) under certain conditions [32]. Thirdly, CSCs have the ability to resistant treatment. CSCs display highly treatment resistance and are responsible for tumour maintenance and tumour recurrence [33]. As reported, CD133-positive glioma stem cells (GCSs) exhibit markedly increased chemotherapy and radiotherapy resistance compared with CD133-negative tumour cells [34]. And high CD44 expressing cancer cells were mostly resistant to drugs [35]. The key mechanism of CSC resistance is that cellular plasticity, especially the ability of CSCs to adopt quiescence, is the key driver [36, 37]. CSCs require input from their specific microenvironment to maintain their properties including but not limited to the above. The roles of these key factors in the CSC microenvironment on CSC properties and tumour development will be detailed in the following sections.

## Extracellular matrix

**Fig. 2:**
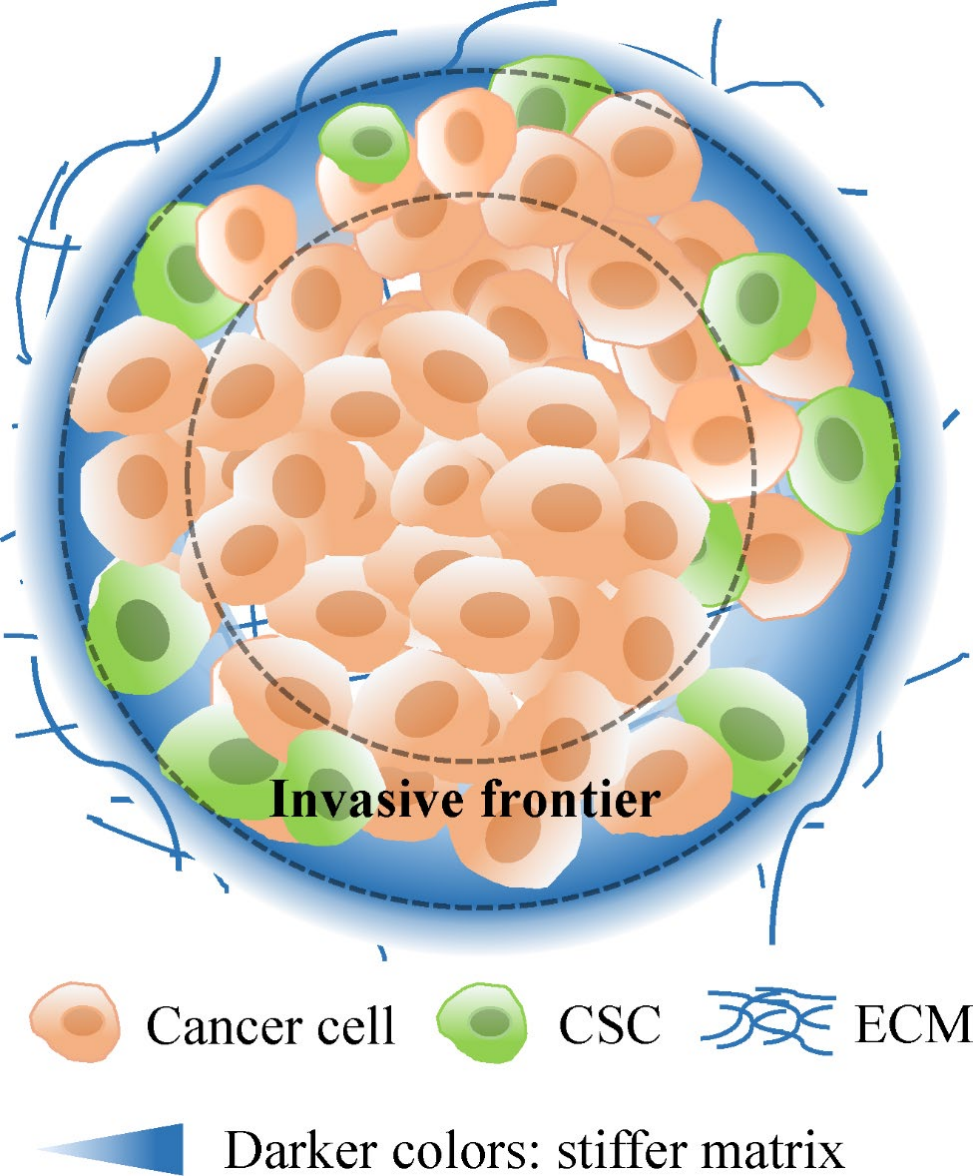
CSC distribution in ECM. Two extending lines from the tumour are drawn to distinguish the invasive frontier area and non-invasive frontier area (internal). CSCs are distributed mainly in the invasive frontier area

ECM is the main structural component of TEM, which comprised of a network of distinct ECM molecules, including collagens, laminins and fibronectin and proteoglycans [38]. It was found that the expression of ECM components such as type I collagen and laminin increased gradually in the radial region from the centre of cancer tissue to the periphery, while the expression of ECM components was almost not detected in the central region of cancer tissue [39]. The addition of type I collagen and laminin leads to an increase in extrinsic matrix stiffness. In fact, the matrix stiffness of cancer tissue increases significantly from the inside out [40, 41]. For cancer tissue, the Young’s modulus in the core area was significantly lower than that in the adjacent normal tissue, but the Young’s modulus in the edge area of the cancer tissue was significantly higher than that in the normal tissue [42]. Some studies have found that the distribution of CSCs is related to this mechanical property of cancer tissue. As reported, aldehyde dehydrogenase 1A1 (ALDH1A1) can serve as a marker for glioma stem cells; moreover, ALDH1A1^+^ cells were increased in the invasive frontier area compared with the non-invasive frontier area [43]. In addition, one study found that the expression of CD133 occurred mainly in the areas close to the tumour rim of HCT116 xenografts, which showed that CD133-positive HCT116 CSCs were distributed mainly in the areas close to the tumour rim [44]. In a study of hepatocellular carcinoma, it was found that the cancer stem cell marker molecules CD133 and CD44 were distributed mainly at the edge of hepatocellular carcinoma stem cell colonies [45]. Moreover, a recent study showed that the highest number of liver CSCs was found at the invasive front part of the tumour [40]. Therefore, as shown in Fig. 2, CSCs with high clonal formation ability and high invasion and metastasis ability gathered mainly in the stiffer invasion frontier area of the cancer tissue.

The accumulation of CSCs in the invasion frontier may come from the transformation of normal/cancer cells or the migration of cancer cells from other regions, which indicates that this region is more conducive to the survival and maintenance of CSC characteristics. Therefore, matrix stiffness in the invasion frontier is an important factor regulating the biological properties of CSCs, such as the maintenance of stemness, invasion, and metastasis. In fact, current studies have demonstrated that the mechanical properties of the cancer microenvironment regulate the expression of CSC markers and associated traits [46, 47]. Mechanical factors, such as matrix stiffness, can influence CSC plasticity, trigger stemness in non-stem cancer cells, [48, 49] and participate in the regulation of biological behaviours [50]. Therefore, mechanical factors, as important factors in cellular and physiological maintenance, play an important role in the regulation of CSC properties and the occurrence and development of cancer.

In addition to matrix stiffness, the prominent components of the ECM, such as type I collagen, fibronectin, and hyaluronan, have also been demonstrated to support the properties of CSCs. Fibronectin and type 1 collagen could increase CSC proliferation and inhibit chemotherapy-imposed apoptosis [51, 52]. Hyaluronan has been proven to support the CSC multipotent state, [53, 54] and depletion of the hyaluronan matrix in vivo decreased CSC marker expression levels in hepatocarcinogenesis [55]. Therefore, CSC fate decisions are dynamically regulated by ECM composition.

## Cellular components of the CSC niche

Stromal cells in CSC niche including TAMs, CAFs, MSCs, MDSCs, ECs, T cells, pericytes etc., play essential roles in the maintenance of CSC function and the occurrence and development of tumors [56]. A large number of studies have proven that CAFs not only secrete a variety of cytokines, growth factors and ECM proteins but are also involved in vascular and lymphatic angiogenesis, ECM remodelling, immunosuppression, and EMT of tumour cells, thus providing a favourable microenvironment for tumour cells, promoting the proliferation, drug resistance, invasion and metastasis of tumour cells, and affecting the prognosis of patients with these tumours [57].

### CAFs

CAFs are a major component of the tumour microenvironment (TME). Numerous evidence demonstrated that CAFs can shape the TME to promote cancer stemness [58]. CAFs can alter the TME, interact with other cell types and support cancer progression through the secretion of soluble factors [59]. Study has shown that CAF-derived cardiotrophin-like cytokine factor 1 (CLCF1) can promote tumour cells to secrete more C-X-C motif chemokine ligand 6 (CXCL6) and transforming growth factor-β (TGF-β), thus promoting the stemness of tumour cells, and in clinical samples, upregulation of the CLCF1-CXCL6/TGF-β axis was significantly associated with an increase in CSCs [60]. CAFs also express vascular endothelial growth factor (VEGF), platelet-derived growth factor (PDGF), interleukin (IL)-8, epidermal growth factor (EGF), and fibroblast growth factor 2 (FGF-2), ultimately forming a tumour growth supporting microenvironment [58, 61]. In addition, CAFs have been shown to promote EMT-driven tumour stemness acquisition and regulate plasticity of lung cancer stemness through paracrine signalling [62, 63]. Therefore, CAFs play important roles in maintaining a favourable tumour microenvironment for tumour development.

### Immune cells

Immune cells, the major cellular components of CSC niche, include TAMs, MDSCs, T cells and so on, which have been proven to play a significant role in tumour development, progression, and therapeutic resistance. TAMs were found to participate in the establishment of the CSC niche through secretory signalling pathway, thus regulating the activity of CSCs [64]. TAMs secrete TNFα and activate NF-κB signalling in CSCs to induce the expression of Slug, Snail, and Twist and consequently drive EMT and CSC self-renewal [65]. In addition, TAMs was reported to regulate murine breast cancer stem cells through a novel paracrine EGFR/Stat3/Sox-2 signalling pathway and promote prostate cancer stem cells self-renewal and prostate cancer metastasis via activating β-catenin/STAT3 signalling [66, 67]. MDSC has also been demonstrated to increase CSC stemness. A study reported the role of MDSCs in the enhancement of breast cancer CSC properties [68]. And a recent study showed that MDSCs increase the stemness and PD-L1 expression of ALDH^High^ ovarian cancer stem cells via the activation of the PI3K/AKT/mTOR signalling pathway [69]. Various populations of T cells exist in tumour at different stages of tumour development, including cytotoxic T cells, regulatory T cells (Tregs), etc. A study showed that ovarian CSCs cooperate with Tregs to promote tumour immune tolerance and enhance tumour progression [70]. Cytotoxic T cells can recognize CSCs in an antigen-specific manner as cancer stem cells express multiple tumour-associated antigens (TAAs), which limits the ability of the adaptive immune system to mount antigen-specific responses to cancer stem cells [71]. In addition to the role of particular immune cell types in driving CSC expansion, the distinct ability of CSCs to evade surveillance and destruction by immune cells also has been demonstrated [72].

### MSCs

MSCs can be recruited to the specific microenvironment by several chemokines, cytokines, growth factors and others produced by tumour cells [73]. MSCs maintain the properties of CSCs mainly by secreting various cytokines, such as CXCL12, IL-6 and IL-8, which can promote the self-renewal of CSCs, [56] and bone morphogenetic protein (BMP) antagonists can maintain the undifferentiated state of CSCs [74]. In addition, MSCs homed at CSC niche play roles by surviving and existing as MSCs or differentiating into another cell type, such as CAFs, macrophages, pericytes or endothelial cells [75, 76]. Evidences indicate that the MSCs can induce EMT and a CSC phenotype in pancreatic cancers and hepatocellular carcinomas, and MSCs increased the stemness of cancer cells in prostate cancer, gastric cancer and ovarian cancer [77].

### ECs

The rapid proliferation of tumour cells increases the size of the tumour and causes the formation of a hypoxic region that activates the tumour to form new blood vessels to provide much-needed nutrients and oxygen. ECs from pre-existing vessels form new blood vessel, that is angiogenesis, plays a key role in cancer growth. EC express VEGF receptor (VEGFR) which bind to VEGF-A, followed by remodelling of the surrounding ECM and formation of new blood vessels [37]. ECs secrete many paracrine factors that directly foster tumour cell proliferation and maintain cancer stem cells [78]. It has been reported that ECs can create a stem cell niche in glioblastoma by providing Notch ligands that nurture self-renewal of CD133-positive cancer stem-like cells [79].

### Pericytes

Pericytes are also important cellular components of the TME. Pericytes have multiple roles in the TEM, including covering ECs along the endothelial surface and participating in basement membrane remodelling and neovascularization during tumorigenesis [80]. Vascular pericytes can be generated by GSCs in vivo, allowing functional blood vessels to promote tumour growth, that suggest the importance of pericytes in remodelling CSC niche [30]. However, the mechanisms are poorly understood and further research is needed.

## Inflammation, hypoxia, and angiogenesis

In the CSC niche, reactions such as inflammation, hypoxia, and angiogenesis constantly occur to keep the specific microenvironment stable, and these biological processes determine the fate of CSCs [81].

### Inflammation

Chronic inflammation is involved in the occurrence, development, invasion, metastasis and other pathological processes of malignant tumours, and it has been found to activate CSCs and cause drug resistance and metastasis [82]. For example, the inflammatory cytokine IL-6 can not only induce the transformation of non-stem cells into CSCs in liver cancer, breast cancer and prostate cancer cell lines but also activate STAT3 signalling to regulate the self-renewal of CSCs [83, 84]. In addition, one study found that the inflammatory factor IKKβ maintains the stemness of cancer cells and promotes metastasis by regulating the LIN28B/TCF7L2 positive feedback loop [85]. Thus, inflammation plays an important role in regulating the biological behaviours of CSCs.

### Hypoxia

Aggressive tumours are known to have hypoxic areas in which cancer cells die from a lack of oxygen [86]. However, for cancer stem cells, the fate is different. Hypoxic regions within tumours probably favour the preservation of the stemness of CSCs [87]. Studies have shown that hypoxic conditions actually promote the properties of CSCs by increasing the expression of hypoxia-inducible factor (HIF) [88, 89]. HIF signalling plays a significant role in the modulation of various signalling pathways (i.e., the Notch, Hedgehog, Hippo, Wnt/β-catenin, and nuclear factor-κB (NF-κB) pathways), which are exploited by CSCs to regulate stemness during hypoxic and therapeutic stress [90, 91]. Meanwhile, HIF signalling enhances the maintenance of a CSC phenotype through the regulation of related genes, including pluripotency-related transcription factors, EMT programmers, glycolysis-associated molecules, drug resistance-associated molecules, miRNAs and VEGF (reviewed in [91]). Therefore, the hypoxic microenvironment plays an important role in maintaining the stemness and function of CSCs. Hypoxia is an important mediator of chemo/radio resistance to cancer therapy through multiple mechanisms. Hypoxia limits radiation therapy efficacy by inhibiting oxygen-mediated free radical damage [92]. In terms of chemotherapy, hypoxia can up-regulate the expression of multidrug resistance-related genes [34].

### Angiogenesis

It is generally believed that tumour angiogenesis plays an important role in tumour recurrence and metastasis. Studies have concluded that there is a strong relationship between CSCs and cancer angiogenesis. On the one hand, CSCs can promote angiogenesis and participate in angiogenesis by secreting a variety of angiogenic factors or directly differentiating into tumour vascular progenitor cells and endothelial cells [93, 94]. Studies have found that CSCs consistently secrete markedly elevated levels of VEGF, and this CSC-mediated VEGF production leads to amplified endothelial cell migration and tube formation in vitro [95]. Another study found that overexpression of VEGF in glioblastoma CSCs induces longer, more vascular and highly destructive tumours [96]. On the other hand, vascular endothelial cells in the tumour microenvironment induce stem cell-like phenotypes in cancer cells and promote the enrichment and migration of CSCs [97, 98]. These results suggest that CSCs promote angiogenesis to form a vascular-rich tumour environment, which in turn is conducive to the maintenance of CSC properties.

## Secretory factors

Cells present in the CSC microenvironment produce several secretory factors that promote CSC properties. Such as cytokines and growth factors, provided by CAFs, MSCs, endothelial cells and specific immune cells, leading to the induction of plasticity, stemness, EMT, and metastasis. The role of TAMs in enhancing and maintaining the stemness of CSC is mainly attributed to their ability to secrete cytokines, chemokines, growth factors and exosomes to enrich the CSC niche [99]. The importance of CAFs in regulating CSC properties discussed above is mainly attributed to the multiple factors they secreted, including pro-angiogenic factors, cytokines (IL-6, TGFβ), chemokines (IL-8, CXC12), prostaglandins (PGE), and growth factors (hepatocyte growth factor (HGF), VEGF) [100]. And, ECs secrete several cytokines such as IL-3, granulocyte colony-stimulating factor (G-CSF), IL-1, IL-6, granulocyte macrophage-CSF, VEGF-A, and basic fibroblast growth factor (bFGF) [101]. In addition to the above, some other cell types in the CSC niche may also secrete factors involved in the maintenance of CSC properties and tumour progression.

## Therapeutic strategies for targeting CSCs and their niche

Studies in recent years have provided important insights into the biological characteristics and maintenance of CSCs. These efforts are also beginning to elucidate potential CSC targeting strategies that could be combined with current treatment strategies to treat cancer more effectively. Cancer stemness is widely accepted as the driving force behind tumour aggressiveness. Researchers have realized that targeting CSCs is of great significance in tumour-targeted diagnosis and treatment. Currently, targeted CSC therapy is carried out mainly with three approaches: induction of CSC differentiation, inhibition of CSC maintenance properties, and targeting of the CSC niche (Table 1).

**Table 1:** Targeted CSC therapy approaches

Approaches	CSC types	Mechanisms	Effect	References
Inducing CSC differentiation	Hepatocellular carcinoma	Smad inhibitor treatment induces CSC differentiation	Tumour growth was suppressed, and 57% of the tumours in a cyclin D1 sphere-derived xenograft model were eliminated	[102]
	Gastric cancer	Targeting phosphoglycerate kinase 1 induces stem cell differentiation in gastric cancer	The invasive potential of gastric cancer cells was impressively reduced in vitro	[103]
	Glioblastoma multiforme	Ciliogenesis induces glioma stem cell differentiation	The infiltration of GSCs into the brain was prevented	[104]
	Breast cancer	ATRA treatment leads to breast cancer stem cell differentiation	Invasion and migration were reduced, and sensitivity to anticancer treatment was increased	[105]
	Glioma	Bone morphogenetic protein 7 induced differentiation of glioma CSCs	Tumour growth, angiogenesis, and invasion were decreased	[106]
Inhibiting CSC maintenance properties	Triple-negative breast cancer	MYC and MCL1 cooperate in the maintenance of chemotherapy-resistant CSCs in TNBC	Tumour initiation was significantly reduced in vivo	[107]
	Prostate and glioblastoma tumours	Suppressing the Wnt signalling pathway	Significant CSC-suppression was induced, and the expression of CSC-related genes was repressed	[108]
	Colon cancer	Honokiol targets notch signalling	CSCs and colon cancer growth were inhibited	[109]
	Non-Small Cell Lung Cancer	NF-κB and MYC signalling are targeted	Inhibition of the cell survival	[110]
	Oesophageal squamous cell carcinoma	Downregulation of ATPase-family AAA-domain-containing protein 2 (ATAD2) inhibits the Hedgehog signalling pathway	The malignant phenotypes of oesophageal squamous cell carcinoma cells were restrained	[11]
Targeting the CSC niche	Breast and Lung cancer	An anti-GPR77 antibody targets CD10^+^GPR77^+^ CAFs, which provide a survival niche for CSCs	Tumour formation was abolished, and tumour chemosensitivity was restored	[111]
	metastatic renal cell carcinoma, hepatocellular carcinoma, gastrointestinal stromal tumours	Antiangiogenic drugs target the VEGF pathway	Successful therapy	[112]

Targeted induction of CSC differentiation is a therapeutic approach that restricts tumour progression by causing loss of the CSC self-renewal capacity and CSC depletion [113]. As reported, the induced differentiation of glioma stem cells (GSCs) by ciliogenesis can prevent the infiltration of GSCs into the brain [104]. In addition, the induction of CSC differentiation reduced drug resistance and invasion ability [114]. The maintenance of CSC properties is usually inhibited by targeting signalling pathways that maintain CSC functions. As reviewed, the “hyaluronan-CD44 axis has a substantial impact on the stemness properties of CSCs and drug resistance, and potential therapeutic approaches targeting CSCs based on the hyaluronan-CD44 axis are also presented” [107]. The Wnt/β-catenin pathway has been reported to facilitate cancer stem cell function maintenance, and compounds inhibit self-renewal and drug resistance of CSCs by targeting the Wnt/β-catenin signalling pathway [115]. The Notch pathway also plays an important role in the maintenance of CSCs by targeting notch signalling, and honokiol inhibits CSCs and colon cancer growth [109]. The Hippo pathway is known to play an important role in tumour progression by regulating various processes, such as cancer cell proliferation, apoptosis, invasion, and metastasis. Abundant evidence has demonstrated the effect of the Hippo pathway on cancer progression based not only on the regulation of cancer cells but also on the regulation of CSCs. Studies have shown the critical role of the Hippo pathway in CSC biology, including in EMT, drug resistance, and self-renewal [116]. In addition, as transcription factors, YAP and TAZ are transcriptional drivers of genes that are essential to the CSC state [12, 48]. Meanwhile, targeting other important signalling pathways (e.g., NF-κB, Hedgehog, and JAK-STAT) in CSCs that maintain their function may also provide strategies for cancer treatment [117, 118].

Inhibiting the maintenance of CSC properties are approaches that have been studied more but have rarely been available in the clinic. As mentioned above, in the CSC niche, a variety of cellular and noncellular components and signalling molecules actively participate in the maintenance of CSC properties. It was observed that dysregulation of pathways that regulate CSC properties often leads to aberrant self-renewal and differentiation of CSCs, which results in carcinogenesis [119, 120]. Signalling pathways, such as Wnt/beta-catenin, Notch, NF-κB and MYC that play key roles in the regulation of CSC properties. For example, by targeting the MYC signalling pathways, tumour initiation of breast cancer reduced significantly in vivo, and cell survival of Non-Small Cell Lung Cancer inhibited [107, 110]. In prostate and glioblastoma tumours, suppressing the Wnt signalling pathway induced significant CSC-suppression and repressed the expression of CSC-related genes [108].

The perfect interaction of CSCs with their niche makes them dynamic and malleable, making them “difficult to target”; therefore, targeting key factors in the CSC niche may be an effective strategy for cancer therapy. In fact, there are a few relevant research results. For example, by targeting CD10^+^GPR77^+^ CAFs, which provide a survival niche for CSCs, an anti-GPR77 antibody abolishes tumour formation and restores tumour chemosensitivity [111]. In addition, recent studies have found that mechanistic stem cell therapy based on the mechanical properties of cancer tissue can precisely target and selectively kill cancer tissue and effectively prevent the toxic side effects caused by cancer radiation and chemotherapy [121]. Inhibitor treatments to block inflammatory cytokines and/or their receptors in CSCs also are effective strategies, as cytokines and their receptors play important roles in the regulation of CSC biological characteristics by changing the cell niche [120]. For example, anti-CD44 antibodies, has been demonstrated to inhibit breast cancer growth, and induce apoptosis, decrease human melanoma metastasis and increase animal survival in SCID mice [28, 122]. Since angiogenesis supports the stemness of CSCs, the regulation of blood vessels is a promising approach to target CSCS for cancer therapy. Indeed, several VEGF-targeting agents have been developed, including bevacizumab, sunitinib, sorafenib, pazopanib, etc [112]. Thus, it is advisable to investigate combined approaches targeting CSCs with factors within the CSC niche that support CSC properties. However, targeting the CSC niche for cancer treatment is only in its infancy and has a long way to go.

## Conclusions and future perspectives

**Fig. 3:**
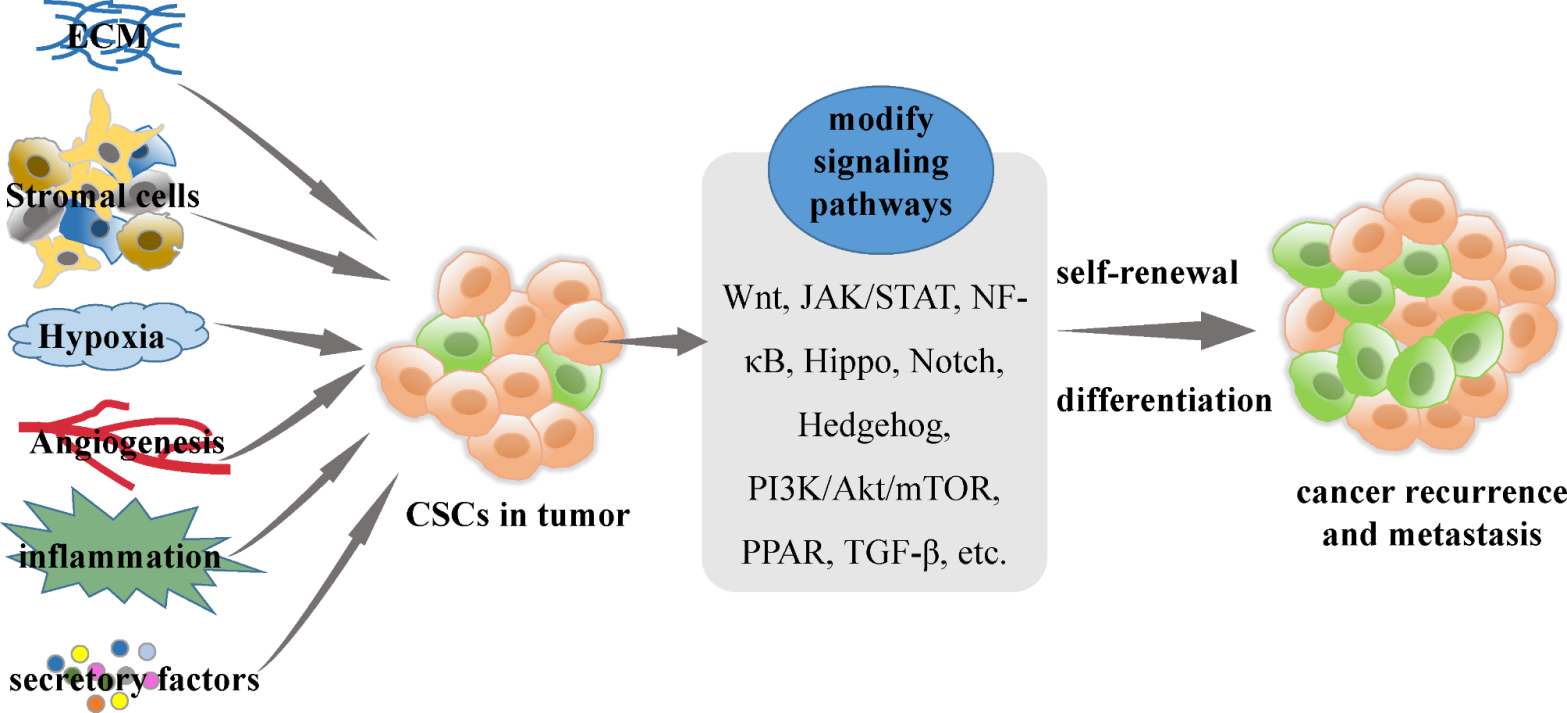
Components of the CSC niche led to cancer recurrence and metastasis by promoting CSC properties. The properties of CSCs in tumours are regulated by components of the CSC niche, including hypoxic regions, inflammatory and immunosuppressive effects, the perivascular compartment, and the ECM. Factors of the CSC niche regulate CSC properties by modifying signalling pathways and ultimately lead to cancer recurrence and metastasis

Multiple factors in the tumour microenvironment play key roles in the management of CSC status (Fig. 3). Here, we have reviewed what is known about the regulation of CSCs by several important CSC niche signals, as well as targeted therapy strategies. Studies have shown that the biological behaviours and functions of CSCs are regulated by a variety of signalling pathways, some of which are triggered by unique properties of the CSC niche. Factors of the CSC niche regulate CSC properties by modifying signalling pathways and ultimately lead to cancer recurrence and metastasis. These factors can affect the conformation and interaction of related molecules in the signalling pathway, triggering biologically important reactions in CSCs that lead to covalent modification of enzymes, protein‒protein interactions, cytoskeletal rearrangement, altered gene expression, and changes in CSC properties. These signalling pathways include the Wnt, NF-κB, Notch, Hedgehog, Hippo, JAK/STAT, PPAR, PI3K/Akt/mTOR, and TGF-β/Smad pathways [117, 123, 124]. In fact, little is known about the regulatory mechanism of multiple factors within the CSC niche on CSC behaviours and characteristics. In the future, multiple innovative strategies should be considered regarding signalling pathways of CSC cross-talk with its niche, which will elucidate potential new approaches for cancer therapy.

## Data Availability

Not applicable.

## Abbreviations

ALDH1A1: Aldehyde dehydrogenase 1A1
bFGF: Basic fibroblast growth factor
BMP: Bone morphogenetic protein
CAF: Cancer-associated fibroblast
CCL: C-C Motif Chemokine Ligand 5
CLCF1: Cardiotrophin like cytokine factor 1
CSC: Cancer stem cell
CXCL: C-X-C Motif Chemokine Ligand
EC: Endothelial cell
ECM: Extracellular matrix
EMT: Epithelial-mesenchymal transformation
FGF-2: Fibroblast growth factor 2
GCS: Glioma stem cell
G-CSF: Granulocyte colony-stimulating factor
HGF: Hepatocyte growth factor
HIF: Hypoxia-inducible factor
IL: Interleukin
MDSC: Myeloid-derived suppressor cell
MSC: Mesenchymal stem cell
NF-κB: Nuclear factor-κB
PDGF: Platelet-derived growth factor
PGE: Prostaglandins
Tregs: Regulatory T cells
TAA: Tumour-associated antigen
TAM: Tumor associated macrophage
TGF-β: Transforming growth factor-β
TIC: Tumour-initiating cell
VEGF: Vascular endothelial growth factor

## References

[1] Chen J, Chen S, Zhuo L, Zhu Y, Zheng H (2020). Regulation of cancer stem cell properties, angiogenesis, and vasculogenic mimicry by miR-450a-5p/SOX2 axis in Colorectal cancer. Cell Death Dis.

[2] Shi C, Kwong DLW, Li X (2022). MAEL augments cancer stemness properties and resistance to sorafenib in hepatocellular carcinoma through the PTGS2/AKT/STAT3 axis. Cancers.

[3] Park HR, Choi YJ, Kim JY, Kim IG, Jung U (2021). Repeated irradiation with γ-ray induces cancer stemness through TGF-β-DLX2 signaling in the A549 human Lung cancer cell line. Int J Mol Sci.

[4] Su Z, Cai L, Lu J (2017). Global expression profile of Tumor stem-like cells isolated from MMQ rat prolactinoma cell. Cancer Cell Int.

[5] Zhang R, Dong M, Tu J (2023). PMN-MDSCs modulated by CCL20 from cancer cells promoted Breast cancer cell stemness through CXCL2-CXCR2 pathway. Signal Transduct Target Ther.

[6] Agliano A, Calvo A, Box C (2017). The challenge of targeting cancer stem cells to halt Metastasis. Semin Cancer Biol.

[7] Tu X, Li C, Sun W (2023). Suppression of cancer cell stemness and drug resistance via MYC destabilization by deubiquitinase USP45 inhibition with a natural small molecule. Cancers.

[8] Plaks V, Kong N, Werb Z (2015). The cancer stem cell niche: how essential is the niche in regulating stemness of Tumor cells?. Cell Stem Cell.

[9] Kreso A, Dick JE (2014). Evolution of the cancer stem cell model. Cell Stem Cell.

[10] Meacham CE, Morrison SJ (2013). Tumour heterogeneity and cancer cell plasticity. Nature.

[11] Li N, Yu Y, Wang B (2020). Downregulation of AAA-domain-containing protein 2 restrains cancer stem cell properties in esophageal squamous cell carcinoma via blockade of the hedgehog signaling pathway. Am J Physiol Cell Physiol.

[12] Jokela TA, LaBarge MA (2021). Integration of mechanical and ECM microenvironment signals in the determination of cancer stem cell states. Curr stem cell Reports.

[13] Mercurio L, Cecchetti S, Ricci A (2017). Phosphatidylcholine-specific phospholipase C inhibition down- regulates CXCR4 expression and interferes with proliferation, invasion and glycolysis in glioma cells. PLoS ONE.

[14] Tatullo M, Marrelli B, Benincasa C (2020). Organoids in translational oncology. J Clin Med.

[15] Oshimori N (2020). Cancer stem cells and their niche in the progression of squamous cell carcinoma. Cancer Sci.

[16] Yoshida GJ, Saya H (2016). Therapeutic strategies targeting cancer stem cells. Cancer Sci.

[17] Oshimori N, Guo Y, Taniguchi S (2021). An emerging role for cellular crosstalk in the cancer stem cell niche. J Pathol.

[18] Aramini B, Masciale V, Grisendi G (2022). Dissecting Tumor growth: the role of cancer stem cells in drug resistance and recurrence. Cancers.

[19] Bhat V, Allan AL, Raouf A (2019). Role of the microenvironment in regulating normal and cancer stem cell activity: implications for Breast cancer progression and therapy response. Cancers.

[20] Chang PH, Sekine K, Chao HM, Hsu SH, Chern E (2017). Chitosan promotes cancer progression and stem cell properties in association with wnt signaling in colon and hepatocellular carcinoma cells. Sci Rep.

[21] Park SY, Lee DG, Jo A (2019). Extracellular microenvironmental change by B16F10 melanoma-derived proteins induces cancer stem-like cell properties from NIH3T3 cells. Sci Rep.

[22] Dzobo K, Senthebane DA, Rowe A (2016). Cancer stem cell hypothesis for therapeutic innovation in clinical oncology? Taking the root out, not chopping the leaf. OMICS.

[23] Atashzar MR, Baharlou R, Karami J (2020). Cancer stem cells: a review from origin to therapeutic implications. J Cell Physiol.

[24] Clarke MF (2019). Clinical and therapeutic implications of cancer stem cells. N Engl J Med.

[25] Kumar VE, Nambiar R, De Souza C, Nguyen A, Chien J, Lam KS (2022). Targeting epigenetic modifiers of Tumor plasticity and cancer stem cell behavior. Cells.

[26] Aikins ME, Qin Y, Dobson HE (2022). Cancer stem cell antigen nanodisc cocktail elicits anti-tumor immune responses in Melanoma. J Control Release off J Control Release Soc.

[27] Lv M, Gong Y, Liu X (2023). CDK7-YAP-LDHD axis promotes D-lactate elimination and ferroptosis defense to support cancer stem cell-like properties. Signal Transduct Target Ther.

[28] Zhang D, Tang DG, Rycaj K (2018). Cancer stem cells: regulation programs, immunological properties and immunotherapy. Semin Cancer Biol.

[29] Tang S, Xiang T, Huang S (2016). Ovarian cancer stem-like cells differentiate into endothelial cells and participate in Tumor angiogenesis through autocrine CCL5 signaling. Cancer Lett.

[30] Cheng L, Huang Z, Zhou W (2013). Glioblastoma stem cells generate vascular pericytes to support vessel function and Tumor growth. Cell.

[31] Auzmendi-Iriarte J, Otaegi-Ugartemendia M, Carrasco-Garcia E (2022). Chaperone-mediated autophagy controls proteomic and transcriptomic pathways to maintain glioma stem cell activity. Cancer Res.

[32] Huang Z, Wu T, Liu AY, Ouyang G (2015). Differentiation and transdifferentiation potentials of cancer stem cells. Oncotarget.

[33] Abdalla Y, Luo M, Mäkilä E, Day BW, Voelcker NH, Tong WY (2021). Effectiveness of porous silicon nanoparticle treatment at inhibiting the migration of a heterogeneous glioma cell population. J Nanobiotechnol.

[34] Biserova K, Jakovlevs A, Uljanovs R, Strumfa I (2021). Cancer stem cells: significance in origin, pathogenesis and treatment of glioblastoma. Cells.

[35] Dzobo K, Sinkala M (2021). Cancer stem cell marker CD44 plays multiple key roles in human cancers: immune suppression/evasion, drug resistance, epithelial-mesenchymal transition, and Metastasis. OMICS.

[36] Batlle E, Clevers H (2017). Cancer stem cells revisited. Nat Med.

[37] Dzobo K, Senthebane DA, Ganz C, Thomford NE, Wonkam A, Dandara C (2020). Advances in therapeutic targeting of cancer stem cells within the Tumor microenvironment: an updated review. Cells.

[38] Dzobo K, Dandara C (2023). The extracellular matrix: its composition, function, remodeling, and role in tumorigenesis. Biomimetics.

[39] Plodinec M, Loparic M, Monnier CA (2012). The nanomechanical signature of Breast cancer. Nat Nanotechnol.

[40] Sun Y, Li H, Chen Q, Luo Q, Song G (2021). The distribution of Liver cancer stem cells correlates with the mechanical heterogeneity of Liver cancer tissue. Histochem Cell Biol.

[41] Wang C, Jiang X, Huang B (2021). Inhibition of matrix stiffness relating integrin β1 signaling pathway inhibits Tumor growth in vitro and in hepatocellular cancer xenografts. BMC Cancer.

[42] Plodinec M, Loparic M, Monnier CA, Obermann EC, Schoenenberger CA (2012). The nanomechanical signature of Breast cancer. Nat Nanotechnol.

[43] Xu SL, Liu S, Cui W (2015). Aldehyde dehydrogenase 1A1 circumscribes high invasive glioma cells and predicts poor prognosis. Am J Cancer Res.

[44] Jin ZH, Sogawa C, Furukawa T (2012). Basic studies on radioimmunotargeting of CD133-positive HCT116 cancer stem cells. Mol Imaging.

[45] Zheng YW, Tsuchida T, Shimao T (2014). The CD133^+^CD44^+^ precancerous subpopulation of oval cells is a therapeutic target for hepatocellular carcinoma. Stem Cells Dev.

[46] Li CS, Vu TL, Luo JJ (2017). Tissue elasticity bridges cancer stem cells to the Tumor microenvironment through microRNAs: implications for a watch-and-wait approach to cancer. Curr Stem Cell Res Ther.

[47] Roy Choudhury A, Gupta S, Chaturvedi PK, Kumar N, Pandey D (2019). Mechanobiology of cancer stem cells and their niche. Cancer Microenviron.

[48] Pankova D, Jiang Y, Chatzifrangkeskou M (2019). RASSF1A controls tissue stiffness and cancer stem-like cells in lung adenocarcinoma. EMBO J.

[49] Safaei S, Sajed R, Shariftabrizi A (2023). Tumor matrix stiffness provides fertile soil for cancer stem cells. Cancer Cell Int.

[50] Northey JJ, Przybyla L, Weaver VM (2017). Tissue force programs cell fate and Tumor aggression. Cancer Discov.

[51] Yu Q, Xue Y, Liu J, Xi Z, Li Z, Liu Y (2018). Fibronectin promotes the malignancy of glioma stem-like cells via modulation of cell adhesion, differentiation, proliferation and chemoresistance. Front Mol Neurosci.

[52] Wu X, Cai J, Zuo Z, Li J (2019). Collagen facilitates the Colorectal cancer stemness and Metastasis through an integrin/PI3K/AKT/Snail signaling pathway. Biomed Pharmacother.

[53] Bourguignon LYW, Earle C, Shiina M (2017). Activation of matrix hyaluronan-mediated CD44 signaling, epigenetic regulation and chemoresistance in Head and Neck cancer stem cells. Int J Mol Sci.

[54] Vaidyanath A, Mahmud H, Khayrani A (2017). Hyaluronic acid mediated enrichment of CD44 expressing glioblastoma stem cells in U251MG xenograft mouse model. J Stem Cell Res Ther.

[55] Sukowati CHC, Anfuso B, Fiore E (2019). Hyaluronic acid inhibition by 4-methylumbelliferone reduces the expression of cancer stem cells markers during hepatocarcinogenesis. Sci Rep.

[56] Zhu P, Fan Z (2018). Cancer stem cells and tumorigenesis. Biophys Rep.

[57] Chen Y, McAndrews KM, Kalluri R (2021). Clinical and therapeutic relevance of cancer-associated fibroblasts. Nat Rev Clin Oncol.

[58] Dzobo K, Senthebane DA, Dandara C (2023). The Tumor microenvironment in tumorigenesis and therapy resistance revisited. Cancers.

[59] Wang Z, Yang Q, Tan Y (2021). Cancer-associated fibroblasts suppress cancer development: the other side of the coin. Front cell Dev Biol.

[60] Lin Y, Cai Q, Chen Y (2022). CAFs shape myeloid-derived suppressor cells to promote stemness of intrahepatic cholangiocarcinoma through 5-lipoxygenase. Hepatology.

[61] Gladkova N, Umezu T, Imanishi S, Kawana C, Ohyashiki JH, Ohyashiki K (2020). Effect of the extracellular component of bone marrow mesenchymal stromal cells from healthy donors on hematologic Neoplasms and their angiogenesis. Hum Cell.

[62] Deng L, Jiang N, Zeng J, Wang Y, Cui H (2021). The versatile roles of cancer-associated fibroblasts in Colorectal cancer and therapeutic implications. Front cell Dev Biol.

[63] Xu DD, Wang Y, Zhou PJ (2018). The IGF2/IGF1R/Nanog signaling pathway regulates the proliferation of acute Myeloid Leukemia stem cells. Front Pharmacol.

[64] Lu H, Clauser KR, Tam WL (2014). A Breast cancer stem cell niche supported by juxtacrine signalling from monocytes and macrophages. Nat Cell Biol.

[65] Liu SJ, Horlbeck MA, Cho SW (2017). CRISPRi-based genome-scale identification of functional long noncoding RNA loci in human cells. Science.

[66] Yang J, Liao D, Chen C (2013). Tumor-associated macrophages regulate murine Breast cancer stem cells through a novel paracrine EGFR/Stat3/Sox-2 signaling pathway. Stem Cells.

[67] Huang R, Wang S, Wang N (2020). CCL5 derived from tumor-associated macrophages promotes Prostate cancer stem cells and Metastasis via activating β-catenin/STAT3 signaling. Cell Death Dis.

[68] Peng D, Tanikawa T, Li W (2016). Myeloid-derived suppressor cells endow stem-like qualities to Breast cancer cells through IL6/STAT3 and NO/NOTCH cross-talk signaling. Cancer Res.

[69] Komura N, Mabuchi S, Shimura K (2020). The role of myeloid-derived suppressor cells in increasing cancer stem-like cells and promoting PD-L1 expression in epithelial Ovarian cancer. Cancer Immunol Immunother.

[70] You Y, Li Y, Li M (2018). Ovarian cancer stem cells promote tumour immune privilege and invasion via CCL5 and regulatory T cells. Clin Exp Immunol.

[71] Vahidian F, Duijf PHG, Safarzadeh E, Derakhshani A, Baghbanzadeh A, Baradaran B (2019). Interactions between cancer stem cells, immune system and some environmental components: friends or foes?. Immunol Lett.

[72] Miranda A, Hamilton PT, Zhang AW (2019). Cancer stemness, intratumoral heterogeneity, and immune response across cancers. Proc Natl Acad Sci.

[73] Kitzberger C, Spellerberg R, Morath V (2022). The sodium iodide symporter (NIS) as theranostic gene: its emerging role in new imaging modalities and non-viral gene therapy. EJNMMI Res.

[74] Davis H, Irshad S, Bansal M (2015). Aberrant epithelial GREM1 expression initiates colonic tumorigenesis from cells outside the stem cell niche. Nat Med.

[75] Schweizer R, Tsuji W, Gorantla VS, Marra KG, Rubin JP, Plock JA (2015). The role of adipose-derived stem cells in Breast cancer progression and Metastasis. Stem Cells Int.

[76] Kitaeva KV, Chulpanova DS, Zhuravleva MN (2022). Characteristics and resistance to cisplatin of human neuroblastoma cells co-cultivated with immune and stromal cells. Bioeng.

[77] Dai X, Zhu K (2023). Cold atmospheric plasma: novel opportunities for Tumor microenvironment targeting. Cancer Med.

[78] Quante M, Varga J, Wang TC, Greten FR (2013). The gastrointestinal Tumor microenvironment. Gastroenterology.

[79] Zhu TS, Costello MA, Talsma CE (2011). Endothelial cells create a stem cell niche in glioblastoma by providing NOTCH ligands that nurture self-renewal of cancer stem-like cells. Cancer Res.

[80] Barachini S, Ghelardoni S, Madonna R. Vascular progenitor cells: from cancer to tissue repair. J Clin Med. 2023;12(6). 10.3390/jcm12062399. 10.3390/jcm12062399 PMC10059009 36983398

[81] Kwon MJ, Shin YK (2013). Regulation of Ovarian cancer stem cells or tumor-initiating cells. Int J Mol Sci.

[82] Shigdar S, Li Y, Bhattacharya S (2014). Inflammation and cancer stem cells. Cancer Lett.

[83] van der Zee M, Sacchetti A, Cansoy M (2015). IL6/JAK1/STAT3 signaling blockade in endometrial cancer affects the ALDHhi/CD126^+^ stem-like component and reduces Tumor burden. Cancer Res.

[84] Wan S, Zhao E, Kryczek I (2014). Tumor-associated macrophages produce interleukin 6 and signal via STAT3 to promote expansion of human hepatocellular carcinoma stem cells. Gastroenterology.

[85] Chen C, Cao F, Bai L (2015). IKKβ enforces a LIN28B/TCF7L2 positive feedback loop that promotes cancer cell stemness and Metastasis. Cancer Res.

[86] Lai F, Liu Q, Liu X, Ji M, Xie P, Chen X (2016). LXY6090 - a novel manassantin a derivative - limits Breast cancer growth through hypoxia-inducible factor-1 inhibition. Onco Targets Ther.

[87] Das B, Tsuchida R, Malkin D, Koren G, Baruchel S, Yeger H (2008). Hypoxia enhances Tumor stemness by increasing the invasive and tumorigenic side population fraction. Stem Cells.

[88] Pang MF, Siedlik MJ, Han S, Stallingsmann M, Radisky DC, Nelson CM (2016). Tissue stiffness and hypoxia modulate the integrin-linked kinase ILK to control Breast cancer stem-like cells. Cancer Res.

[89] Chang WH, Lai AG (2019). Aberrations in Notch-Hedgehog signalling reveal cancer stem cells harbouring conserved oncogenic properties associated with hypoxia and immunoevasion. Br J Cancer.

[90] Guo Y, Xiao Z, Yang L (2020). Hypoxia–inducible factors in hepatocellular carcinoma. Oncol Rep.

[91] Emami Nejad A, Najafgholian S, Rostami A (2021). The role of hypoxia in the Tumor microenvironment and development of cancer stem cell: a novel approach to developing treatment. Cancer Cell Int.

[92] Jiang Y, Verbiest T, Devery AM (2016). Hypoxia potentiates the radiation-sensitizing effect of olaparib in human non-small cell Lung cancer xenografts by contextual synthetic lethality. Int J Radiat Oncol Biol Phys.

[93] Zhu Z, Xu J, Li L (2020). Effect of gastric cancer stem cell on gastric cancer invasion, migration and angiogenesis. Int J Med Sci.

[94] Nawara HM, Afify SM, Hassan G (2021). An assay for cancer stem cell-induced angiogenesis on chick chorioallantoic membrane. Cell Biol Int.

[95] Folkins C, Shaked Y, Man S (2009). Glioma Tumor stem-like cells promote Tumor angiogenesis and vasculogenesis via vascular endothelial growth factor and stromal-derived factor 1. Cancer Res.

[96] Khasraw M, Fujita Y, Lee-Chang C, Balyasnikova IV, Najem H, Heimberger AB (2022). New approaches to glioblastoma. Annu Rev Med.

[97] Schwitalla S, Fingerle AA, Cammareri P (2013). Intestinal tumorigenesis initiated by dedifferentiation and acquisition of stem-cell-like properties. Cell.

[98] Sun L, Wang Y, Wang L (2019). Resolvin D1 prevents epithelial-mesenchymal transition and reduces the stemness features of hepatocellular carcinoma by inhibiting paracrine of cancer-associated fibroblast-derived COMP. J Exp Clin Cancer Res.

[99] Mishra AK, Banday S, Bharadwaj R, et al. Macrophages as a potential immunotherapeutic target in solid cancers. Vaccines. 2022;11(1). 10.3390/vaccines11010055. 10.3390/vaccines11010055 PMC9863216 36679900

[100] Wilczyński JR, Wilczyński M, Paradowska E (2022). Cancer stem cells in ovarian cancer-a source of Tumor success and a challenging target for novel therapies. Int J Mol Sci.

[101] Schwerdtfeger M, Desiderio V, Kobold S, Regad T, Zappavigna S, Caraglia M (2021). Long non-coding RNAs in cancer stem cells. Transl Oncol.

[102] Xia W, Lo CM, Poon RYC (2017). Smad inhibitor induces CSC differentiation for effective chemosensitization in cyclin D1- and TGF-β/Smad-regulated Liver cancer stem cell-like cells. Oncotarget.

[103] Zieker D, Bühler S, Ustündag Z (2013). Induction of Tumor stem cell differentiation–novel strategy to overcome therapy resistance in gastric cancer. Langenbeck’s Arch Surg.

[104] Goranci-Buzhala G, Mariappan A, Ricci-Vitiani L (2021). Cilium induction triggers differentiation of glioma stem cells. Cell Rep.

[105] Yan Y, Li Z, Xu X (2016). All-trans retinoic acids induce differentiation and sensitize a radioresistant Breast cancer cells to chemotherapy. BMC Complement Altern Med.

[106] Lee J, Son MJ, Woolard K (2008). Epigenetic-mediated dysfunction of the bone morphogenetic protein pathway inhibits differentiation of glioblastoma-initiating cells. Cancer Cell.

[107] Lee KM, Giltnane JM, Balko JM (2017). MYC and MCL1 cooperatively promote chemotherapy-resistant Breast cancer stem cells via regulation of mitochondrial oxidative phosphorylation. Cell Metab.

[108] Arrillaga-Romany I, Odia Y, Prabhu VV (2020). Biological activity of weekly ONC201 in adult recurrent glioblastoma patients. Neuro Oncol.

[109] Ponnurangam S, Mammen JMV, Ramalingam S (2012). Honokiol in combination with radiation targets notch signaling to inhibit colon Cancer stem cells. Mol Cancer Ther.

[110] Windmöller BA, Beshay M, Helweg LP (2021). Novel primary human cancer stem-like cell populations from non-small cell Lung cancer: inhibition of cell survival by targeting NF-κB and MYC signaling. Cells.

[111] Su S, Chen J, Yao H (2018). CD10(+)GPR77(+) cancer-associated fibroblasts promote cancer formation and chemoresistance by sustaining cancer stemness. Cell.

[112] Ebos JML, Kerbel RS (2011). Antiangiogenic therapy: impact on invasion, Disease progression, and Metastasis. Nat Rev Clin Oncol.

[113] Garg M (2009). Gain of antitumor functions and induction of differentiation in cancer stem cells contribute to complete cure and no relapse. Crit Rev Oncog.

[114] Lee IC, Fadera S, Liu HL (2019). Strategy of differentiation therapy: effect of dual-frequency ultrasound on the induction of Liver cancer stem-like cells on a HA-based multilayer film system. J Mater Chem B.

[115] Zhang Y, Wang X (2020). Targeting the Wnt/β-catenin signaling pathway in cancer. J Hematol Oncol.

[116] Park JH, Shin JE, Park HW (2018). The role of hippo pathway in cancer stem cell Biology. Mol Cells.

[117] Yang L, Shi P, Zhao G (2020). Targeting cancer stem cell pathways for cancer therapy. Signal Transduct Target Ther.

[118] Ju F, Atyah MM, Horstmann N (2022). Characteristics of the cancer stem cell niche and therapeutic strategies. Stem Cell Res Ther.

[119] Chaudhuri A, Kumar DN, Dehari D (2022). Emergence of nanotechnology as a powerful cavalry against triple-negative Breast cancer (TNBC). Pharmaceuticals.

[120] Zeng X, Liu C, Yao J (2021). Breast cancer stem cells, heterogeneity, targeting therapies and therapeutic implications. Pharmacol Res.

[121] Yang JD, Nakamura I, Roberts LR (2011). The Tumor microenvironment in hepatocellular carcinoma: current status and therapeutic targets. Semin Cancer Biol.

[122] Lv L, Shi Y, Wu J, Li G (2021). Nanosized drug delivery systems for Breast cancer stem cell targeting. Int J Nanomedicine.

[123] Clara JA, Monge C, Yang Y, Takebe N (2020). Targeting signalling pathways and the immune microenvironment of cancer stem cells - a clinical update. Nat Rev Clin Oncol.

[124] Leon G, MacDonagh L, Finn SP, Cuffe S, Barr MP (2016). Cancer stem cells in drug resistant Lung cancer: targeting cell surface markers and signaling pathways. Pharmacol Ther.

